# Postoperative acute kidney injury requiring renal replacement therapy in cardiac surgical patients: risk and protective factors

**DOI:** 10.1186/1749-8090-10-S1-A283

**Published:** 2015-12-16

**Authors:** F Ampatzidou, L Karagounis, CP Koutsogiannidis, T Karaiskos, A Madesis, A Vlachou, G Drossos

**Affiliations:** 1 Cardiothoracic Surgery Department G. Papanikolaou Hospital Exohi, Thessaloniki, 57010, Greece; 2 Interbalkan Medical Centre of Thessaloniki, 551 33, Greece

## Background/Introduction

Postoperative acute kidney injury (AKI) has negative impact on patient's outcome after cardiac surgical procedures. Reported incidence of AKI requiring renal replacement therapy is approximately 1-3% of cardiac surgical patients and is characterized by extremely high morbidity and mortality rates.

## Aims/Objectives

Aim of our study was to investigate perioperative factors associated with postoperative AKI requiring renal replacement therapy in patients undergone cardiac surgery.

## Method

The study group was consisted of 1186 consecutive patients who underwent cardiac surgery from June 2012 to April 2015 in a single Cardiothoracic Surgery Department. The following 21 peri-operative variables have been investigated: Age, Gender, Euroscore II, Body Mass Index (BMI),Smoking habit, Diabetes Mellitus, Insulin use, pre-op Glomerular Filtration Rate (GFR), Peripheral Vascular Disease (PVD), Chronic obstructive pulmonary disease (COPD), chronic atrial fibrillation, REDO, Angiotensin-converting enzyme inhibitors (ACEi) use, Ejection Fraction, Pulmonary Hypertension, Urgent/emergent operations, Cardio-pulmonary bypass (CPB) time, total red blood cells (RBC) units transfused, mechanical ventilation time, Prolonged inotropic support (>24 hours), Low cardiac output syndrome (LCOS). Factors with statistical significance according to univariate analysis, underwent multivariate logistic regression analysis.

## Results

Univariate analysis revealed the following11 factors having statistical significant relationship with postoperative AKI requiring renal replacement therapy: Euroscore II(p < 0.01), preoperative GFR (p < 0.01), COPD (p = 0,032), ACEi use (p = 0.032), pulmonary hypertension (p = 0.01), emergent/urgent status (p < 0.01), CPB time (p < 0.01), RBC units (p < 0.01), mechanical ventilation time (p < 0.01), Prolonged inotropic support (p < 0.01) and LCOS (p < 0.01). Logistic regression analysis of the above 11 factors revealed that AKI requiring renal replacement therapy is associated with prolonged inotropic support (p < 0.00), total RBC transfused units (p < 0.00), not use of ACEi (p < 0.027).

## Discussion/Conclusion

Prolonged inotropic support and total RBC transfused units are factors associated with renal replacement therapy after cardiac surgery. It seems that the use of ACEi has renal protective properties, avoiding the most serious type of AKI which demands renal replacement therapy.

